# Malondialdehyde value as radical oxidative marker and endogenous antioxidant value analysis in brain tumor

**DOI:** 10.1016/j.amsu.2021.103231

**Published:** 2022-03-23

**Authors:** Ridha Dharmajaya, Dina Keumala Sari

**Affiliations:** a Department of Neurosurgery, Faculty of Medicine Universitas Sumatera Utara, Medan, Indonesia; b Department of Nutrition, Faculty of Medicine Universitas Sumatera Utara, Medan, Indonesia

**Keywords:** Brain tumour, Malondialdehyde, Superoxide dismutase, Endogenous antioxidant, Neurooncology

## Abstract

**Introduction:**

Oxidative stress has been considered as one of many contributor in developing risk of cancer. Oxidative stress may also promote the increasing number of free radical. Malondialdehyde (MDA) is one of radical oxidative marker, while Superoxide Dismutase (SOD) play role as endogenous antioxidant. It has been postulated that in cancer cells there is an increase of oxidative stress compared to normal cell.

**Method:**

This study is a case controlled analytical study to find the relationship between levels of MDA and SOD in patients with brain tumours. The sample obtained was 35 people who met the inclusion and exclusion criteria. Based on this analysis, it will be determined whether there is a significant relationship between levels of MDA and SOD in each type of brain tumours.

**Result:**

There is no significant relationship from all groups brain tumour and all tumours have a low correlation (r = 0.187) in the value of superoxide dismutase level. There is also no significant relationship from all groups (p = 0.302) and a low correlation (r = 0.187) to the value of Malondialdehyde level.

**Discussion:**

There was no relationship between superoxide dismutase in any type of intracranial tumour in this study. These concluded that superoxide values could not be a risk factor for primary intracranial tumours. Levels of MDA which is an indicator of lipid peroxidation, were significantly higher in patients consisting of meningiomas and gliomas. In high grade gliomas, the MDA increases due to the progressive progression of glioma tumours due to an increase in Reactive oxygen species levels.

**Conclusion:**

This study shows no correlation between SOD as an endogenous antioxidant and MDA as radical oxidative marker in primary brain tumour.

## Highlights

• Brain Tumour.
• Malondialdehyde.
• Superoxide Dismutase.
• Endogenous Antioxidant.
• Neurooncology.

## 1 Introduction

There are two state of oxidative stress, such are the formation of reactive oxygen species and antioxidant effect on cell. The formation of oxidative stress require endogenous and exogenous source. The source may be formed from mitochondria and peroxisome [1].

Oxidative stress has been considered as one of many contributor in developing risk of cancer. There is an increase of reactive oxygen species (ROS) in patients with continuous inflammation, and thus may be a recruitment of large amount of activated cells and thus lead to preneoplastic event, and may overcome the level of antioxidant in cell level, and thus may alter the genetic which will lead to increasing oncogene [2].

Oxidative stress may also promote the increasing number of free radical. Malondialdehyde (MDA) is one of free radical, which is formed from the reaction of free radical and lipid and may alter the structure of cell membrane, and later may cause DNA alteration in cell level [3].

Many studies have reported superoxide dismutase (SOD) in cellular level. There are three types of SOD, the cuprum SOD or so called SOD1, located within cytosol, inner membrane mitochondrial and nucleus. SOD2 or called the Manganese SOD as the most significant type of SOD in cellular level. EcSOD (SOD3) predominantly an antioxidant enzyme [4]. Many studies have reported the role of SOD2 in brain tumour, such as gliomas. It has been reported that the SOD2 increased in glioma tissues in comparison to the control tissues [5].

It has been postulated that in cancer cells, there is an increase of oxidative stress compared to normal cell. Brain tumour may be caused by alteration in gene and promoting oncogene. Thus, inflammation still play an important role in oncogenesis in primary brain tumour. This study will observe the relationship of SOD and MDA in brain tumour case.

## 2 Method

This study is a case controlled analytical study to find the relationship between levels of MDA and SOD in patients with brain tumours. Samples were collected from patients at Haji Adam Malik General Hospital in Medan, Indonesia. The patient's blood serum was taken to be checked for these variables.

The sample obtained was 35 people who met the inclusion and exclusion criteria. The sample included are patient willing to be included as sample, have complete medical record and have undergo diagnostic procedure to confirm the diagnosis of brain tumour. Patients over 70 years old, with high comorbidities and with a previous brain surgery are excluded in this study.

Samples were categorized based on demographic data (age and gender), then the samples were categorized based on the type of brain tumour suffered. Once categorized, the levels of MDA and SOD were examined. Specimens were taken from the patient's peripheral blood examination and analyzed in laboratorium. The results of these examinations are grouped on a nominal scale and analyzed statistically.

Analysis using SPSS Program to determine the relationship between this variable. Based on this analysis, it will be determined whether there is a significant relationship between levels of MDA and SOD in brain tumours.

## 3 Result

### 3.1 Sample characteristic

In the demographic results, it was found that the most sample was male as many as 20 people (57%) and the most age was 41–50 years as many as 15 people (43%). This data can be seen in Table 1.

**Table 1:** Sample gender and age.

Description	Parameter	n
Gender	Men	20
	Women	15
	Total	35
Age	<20	3
	21–30	3
	31–40	5
	41–50	15
	51–60	5
	61–70	4
	Total	35

Types of tumours are divided into 3 categories, Meningioma, Glioma and Brain metastases based on Head CT scan images, head MRI with contrast and histopathology. The most common type of tumour was meningioma as many as 15 people (44%). The data is attached in Table 2.

**Table 2:** Type of brain tumour.

Description	Type	n
Type of Brain Tumour	Meningioma	15
	Glioma	10
	Brain Metastasis	10
	Total	35

**Table 3:** GCS and KPS.

No	Description	Parameter	n
1	Glassgow coma Scale (GCS)	3–8	0
		9–12	2
		13–15	33
		Total	35
2	Karnofsky Perfomance Score (KPS)	100	33
		90	2
		80	0
		70	0
		60	0
		50	0
		40	0
		30	0
		20	0
		10	0
		0	0
		Total	35

The sample obtained was then carried out an assessment of the degree of consciousness based on GCS and the value of karnofsky performance status (KPS).

### 3.2 Superoxide Dismutase (SOD) value in sample

**Fig. 1:**
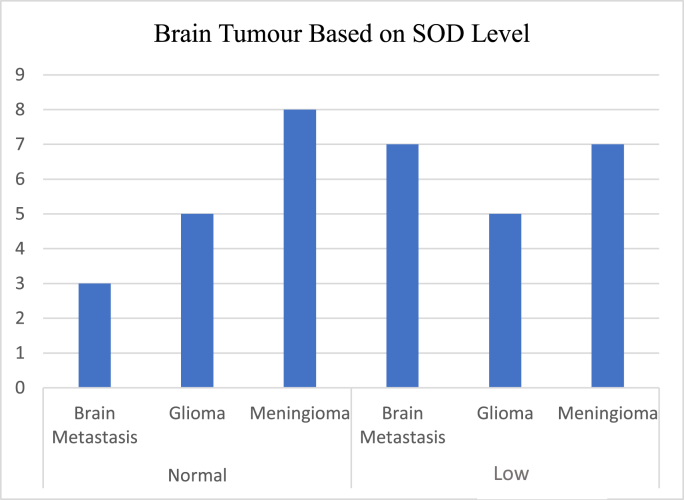
SOD value in brain tumour.

In meningioma tumours, 8 samples (53%) had normal SOD values, while 7 samples (47%) had decreased SOD values. In glioma type tumours, 5 samples (50%) had normal SOD values and 5 samples (50%) had decreased SOD values. In brain metastatic tumours, 7 samples (70%) had decreased SOD values (Fig. 1).

Based on Fig. 1 and Table 4, it can be concluded that in all groups of brain tumours there is no significant relationship with superoxide dismutase (p = 0.66) (see Table 3). Based on the correlation value, all tumours have a low correlation (r = 0.187) to the value of superoxide dismutase level. Between all tumour group using post hoc test there are also no significant correlation between every group regarding SOD level based on Table 5.

**Table 4:** Brain tumour and SOD.

Brain Tumour	Superoxide Dismutase	p	r
Glioma	Normal	0.66 0.66	0.187 0.187
Meningioma	Low		
Brain Metastases

**Table 5:** Tukey HSD test in SOD level between tumour group.

Brain Tumour	SOD level	
	Δ	P value
Meningioma vs glioma	−0.853	0.998 0.669 0.751
Meningioma vs brain metastasis	−1.102	
Glioma vs brain metastasis	−1.1017	

### 3.3 Malondyaldehyde (MDA) value in sample

**Fig. 2:**
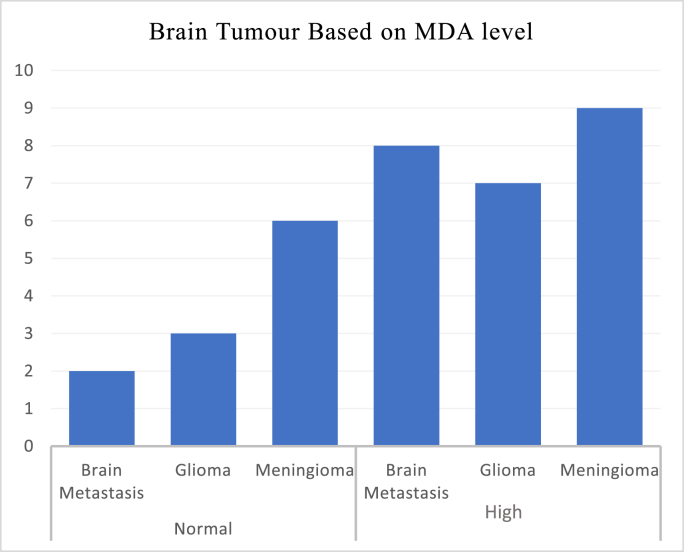
MDA value in brain tumour.

In meningioma tumours, 6 samples (40%) had normal MDA values, while 9 samples (60%) had decreased higher values. In glioma type tumours, 3 samples (30%) had normal MDA values and 7 samples (70%) had higher MDA values. In brain metastatic tumours, 9 samples (90%) had higher MDA values (Fig. 2).

**Fig. 3:**
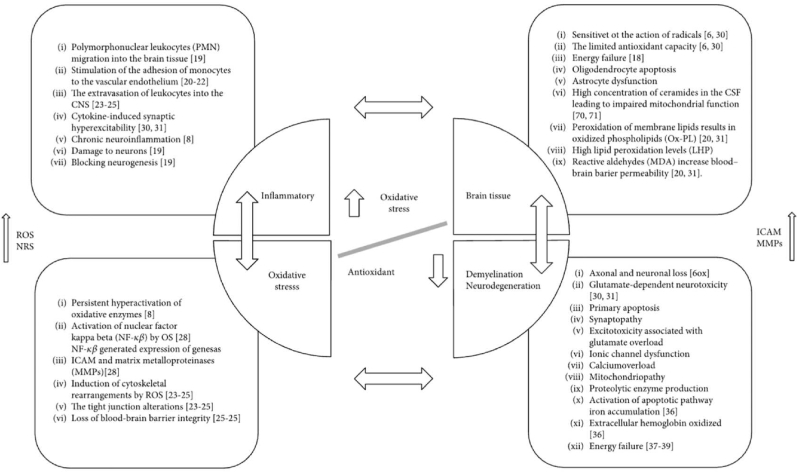
Schematic relationship of antioxidant and oxidative stress in brain tissue [8].

Based on Fig. 2 and Table 6, it can be concluded that in all groups of brain tumours there is no significant relationship from all groups (p = 0.173). Based on the correlation value, all tumours have a low correlation (r = 0.179) in the value of Malondialdehyde level. Between all tumour group using post hoc test there are also no significant correlation between every group regarding MDA level based on Table 5 (see Table 7).

**Table 6:** Brain tumour and MDA.

Brain Tumour	MDA	p	r
Glioma	Normal	0.173 0.173	0.179 0.179
Meningioma	High		
Brain Metastases

**Table 7:** Tukey HSD test in MDA level between tumour group.

Brain tumour	MDA Level	
	Δ	P value
Meningioma vs Glioma	−3,876	0.155
Meningioma vs brain metastasis	−2,992	0.319
Glioma vs brain metastasis	0.878	0.918

## 4 Discussion

Reactive oxygen species (ROS) are important modulators of metabolism, signal transduction and stress responses by acting as secondary messengers for redox sensitive substrates, altering protein structure and function. ROS are spatially restricted due to the limited diffusion distance afforded by the short half-life. Oxidative stress may also promote the increasing number of free radical [4].

Malondialdehyde (MDA) is one of free radical, which is formed from the reaction of free radical and lipid and may alter the structure of cell membrane, and later may cause DNA alteration in cell level [3].

One of the important enzymatic components of the antioxidant defense system is superoxide dismutase (SODs). There are three members of the SOD that exist physiologically, with a tightly regulated localization pattern [4]. The enzyme SOD, which catalyzes the spontaneous dismutation of superoxide radicals to hydrogen peroxide, is present in all parts of the nervous system, including: mitochondrial intermembrane space (SOD1; copper/zinc SOD), mitochondrial matrix (SOD2; manganese SOD), and plasma, lymph, and synovial fluid (SOD3; extracellular SOD). According to Rajaraman, an increase in SOD3 in the blood can be a risk factor for the presence of meningiomas or gliomas, but according to a 2019 study, that there was no relationship between superoxide dismutase and any type of intracranial tumour, such as gliomas and meningiomas, and concluded that superoxide values could not be a factor. Risk for primary intracranial tumours [6,7].

Normalization of SOD levels contributed to the reduction of the cancer cell phenotype. Current research has suggested that SOD may regulate cancer progression and, therefore, may be used as a new target for cancer treatment. SOD liposomes/mimetics have experimentally shown promising results to produce animal models of cancer prevention [9].

It has been conceptualized that the overexpression of SOD may inhibit cell proliferation, and thus the decrease of SOD may promote the tumoural cell growth. This study found no correlation between SOD and brain tumour in patients. A study stated that perturbation of SOD level may favour tumour growth [10].

Brain tissue has many fatty acid, predominantly arachidonic acid and decosahexaenoic acid, and perform as main structure of lipid membrane. The enzymatic metabolism may produce proinflammatory mediator, and may lead to production of ROS thus may increase the level of lipid peroxidation [11].

In the study by Yilmaz N, levels of MDA, which is an indicator of lipid peroxidation, were significantly higher in patients consisting of meningiomas and gliomas compared to healthy controls. In another study, the MDA level in gliomas increased with tumour grading. In high grade gliomas, the MDA increases due to the progressive progression of glioma tumours due to an increase in ROS levels [12]. In this study, there is no correlation between MDA level in primary brain tumour, and, may be caused by fewer grade of brain tumour in our institution, opposite to the study which stated that higher grade glioma may increase MDA level. Also, a study from Wozniak B et al. stated that higher level of MDA was found in brain tumour patient, as a result of higher level of lipid peroxidation in central nervous system tumour group [13]. Another study also stated that the level of lipid peroxidation is lower in adjacent peritumoral tissue, which from the study stated that glioma has higher level than meningioma level and brain metastasis level [14]. A study from Cirak et al. stated that the level of MDA is also correlate with the grade of brain tumour [15].

## 5 Conclusion

This study shows no correlation between SOD and MDA in primary brain tumour. This correlates with another study that stated there is no increase in oxidative stress and primary brain tumour, caused by lower glioma grade in our institution.

## Ethical Approval

Approval has been given by ethical committee of Universitas Sumatera Utara

## Consent

Not Applicable

## Sources of funding

None

## Author contribution

Ridha Dharmajaya : Author

Dina Keumala Sari: Co- Author

## Registration of Research Studies

Name of the registry: None

Unique Identifying number or registration ID: None

Hyperlink to your specific registration (must be publicly accessible and will be checked): None

## Guarantor

Ridha Dharmajaya: Author

Email : dharmajayaridha@gmail.com

## Declaration of competing interest

There is no conflict of interest in this study
